# Supplementary material for: Prognostic Value of the Immunological Subtypes of Adolescent and Adult T-Cell Lymphoblastic Lymphoma; an Ultra-High-Risk Pro-T/CD2(−) Subtype
Source: Cancers (Basel). 2021 Apr 15;13(8):1911. doi: 10.3390/cancers13081911 (PMC8071379; doi:10.3390/cancers13081911)
Supplement: Supplementary file 1 [file cancers-13-01911-s001.zip › cancers-1157524-conversion supplementary.pdf]

cancers MDPI

Supplementary Materials: Prognostic Value of the Immunological Subtypes of Adolescent and Adult T-Cell Lymphoblastic Lymphoma, an Ultra-High-Risk Pro-T/CD2(−) Subtype

Beata Ostrowska, Grzegorz Rymkiewicz, Magdalena Chechlinska, Katarzyna Blachnio, Katarzyna Domanska-Czyz, Zbigniew Bystydzienski, Joanna Romejko-Jarosinska, Anita Borysiuk, Sebastian Rybski, Wojciech Michalski and Jan Walewski

Table S1A. A minimal panel of anti-human monoclonal antibodies (mAbs) used for the flow cytometry (FCM) analysis.

mAb	Fluorochrome	Clone	Source
CD1a	PE	SK9	BD[1]
CD2	FITC, PE, APC	S5.2	BD
CD3	FITC, PE, PerCP, APC, APC-H7, APC-Cy7	SK7	BD
cCD3/CD79α/MPO *	FITC/PE/PerCP	UCHT1/5B8/HM47	BD Oncomark
CD4	FITC, APC	SK3	BD
CD5	FITC, PE, APC	L17F12	BD
CD7	FITC, PE, APC	M-T701	BD
CD8	FITC, PE, PE-Cy7, APC	SK1	BD
CD13	PE	L138	BD
CD15	FITC	MMA	BD
CD33	APC	P67.6	BD
CD34	FITC, PE	8G12	BD
TdT *	FITC	E17-1519	BD
HLADR	FITC, PerCP	L243	BD

Table S1B. Additional mAbs used for extended FCM diagnostics.

CD3/HLADR	FITC/PE	SK7/L243	BD Simultest
CD45/CD14	FITC/PE	2D1,24/MφP9	BD Simultest
CD3/CD16&56	FITC/PE	SK7/B73.1/MY31	BD Simultest
CD3/CD19	FITC/PE	SK7/4G7	BD Simultest
CD4/CD8/CD3	FITC/PE/PerCP	SK3/SK1/SK7	BD Tritest
CD3/CD8/CD45/CD4	FITC/PE/PerCP/APC	SK7/SK1/2D1/SK3	BD Multitest
CD5/CD19	FITC/PE	L17F12/SJ25C1	BD Simultest
CD5/CD10/CD19	FITC/PE/PerCP-Cy5	L17F12/HI10a/SJ25C1	BD Oncomark
CD10	PE, PE-Cy7	HI10a	BD
CD25	PE, PE-Cy7, APC	2A3	BD
CD26	FITC	L272	BD
CD27	APC	L128	BD
CD28	APC, APC-H7	CD28.2	BD Pharmingen
CD38	FITC, PE, APC	HB-7	BD
CD43	APC	L10	ThermoFisher Scientific[2]
CD44	PE	MEM-85	ThermoFisher Scientific
CD45	FITC, Per-CP	2D1	BD
CD45RA	FITC	L48	BD
CD45RO	PE	UCHL1	BD
CD49d	APC	9F10	BD Pharmingen
CD52	PE	4C8	BD Pharmingen
CD54	APC	HA58	BD Pharmingen
CD56	FITC, PE, APC	NCAM16.2	BD

CD62L	PE	SK11	BD
CD71	FITC, APC	L01.1	BD
CD81	APC	JS-81	BD Pharmingen
CD94	APC	HP-3D9	BD
CD200	PE	MRC OX-104	BD Pharmingen
CD305	PE	DX26	BD Pharmingen
TCRα/β	FITC	WT31	BD
TCRγ/δ	PE	11F2	BD
IOTest BetaMark TCRVb Repertoire Kit	PE, PE+FITC, FITC	24 different clones	Beckman Coulter[3]
IgG1/IgG2a	FITC/PE	X40/X39	BD Simultest
IgG1	APC	MOPC-21	BD

Abbreviations: mAb, monoclonal antibodies; FITC, fluorescein isothiocyanate; PE, phycoerythrin; PerCP, peridinin-chlorophyll protein; PerCP-Cy5.5, PerCP with a cyanine dye (Cy5.5); PE-Cy7, PE with a cyanine dye (Cy7); APC, allophycocyanin; IgG1/IgG2a and IgG1, antibodies of isotype control conjugated with FITC/PE and APC, respectively. *The intracytoplasmic staining of CD3, CD79α and MPO, and intranuclear staining of TdT was performed following permeabilization of cells with PermeaFix/Perm2 (BD—Becton Dickinson, San Jose, CA, USA.). [1] BD, Becton Dickinson, San Jose, CA, USA; [2] ThermoFisher Scientific, Waltham, MA, USA; [3] Beckman Coulter, manufactured by Immunotech SA, Marseille, Cedex.

Table S2. Details of chemotherapy GMALL 05/93 (**A**) and GMALL T-LBL 1/2004 (**B**). **(A)** GMALL 05/93 Total treatment duration in T-ALL was 2.5 years (with maintenance therapy), in T-LBL at least 6 months (including reinduction) and up to 12 months was recommended. Central nervous system irradiation (24 Gy), after CR confirmation and mediastinal irradiation (36 Gy) was recommended during the second induction.

Drug	Dose	Weeks	Days of Administration
	**Induction I**		
Prednisone	60 mg/m^2/p.o.[1]	1–4	d.[4]1–28 (than taper over few days)
Vincristine	2 mg total/i.v.[2]		d. 1, 8, 15, 22
Daunorubicin	45 mg/m^2/i.v.		d. 1, 8, 15, 22
Methotrexate (MTX)/ Dexamethasone (Dx)	15/4 mg i.th.[3]		d. 1
L-asparaginase	5000 IU/m^2/i.v.		d.15–28 (every other day)
	**Induction II**		
Cyclophosphamide	1000 mg/m^2/i.v.	5–8	d. 29, 43, 57
Cytarabine (Ara-C)	75 mg/m^2/i.v.		d. 31–34, 38–41, 45–48, 52–55
6-Mercaptopurine	60 mg/m^2/p.o.		d. 29–57
MTX/Dx	15/4 mg i.th.		d. 31, 38, 45, 52
Mediastinal irradiation (24 Gy) during phase II of induction (all patients) and prophylactic cranial irradiation (24 Gy) after achievement of CR			
	**Consolidation I/II**		
Cytarabine	1000 mg/m^2/i.v. (every 12 hours)	13–14	d. 1–4
Mitoxantrone	10 mg/m^2/i.v.		d. 3–5
Methotrexate	1500 mg/m^2/i.v. 24 h infusion	17–20	d. 1, 15
L-asparaginase	10000 IU/m^2		d. 2, 16
6-Mercaptopurine	25 mg/m^2		d. 1–5, 15–19

**Reinduction I**			
Prednisone<br>Vincristine<br>Adriamycine<br>MTX/Ara-C/Dx	60 mg/m²/p.o.<br>2 mg total/i.v.<br>25 mg/m²/i.v.<br>15/40/4 mg i.th.	21–24	d. 1–28<br>d. 1, 8, 15, 22<br>d. 1, 8, 15, 22<br>d. 1
**Reinduction II**			
Cyclophosphamide<br>Cytarabine<br>6-Thioguanine<br>MTX/Ara-C/Dx	1000 mg/m²/i.v.<br>75 mg/m²/i.v.<br>60 mg/m²/p.o.<br>15/40/4 mg i.th.	25–27	d. 29<br>d. 31–34, 38–41<br>d. 29–42<br>d. 29
**Consolidation III/IV/V/VI**			
Cyclophosphamide<br>Cytarabine	1000 mg/m²/i.v.<br>500 mg/m²/i.v. 24 h	33–52	d. 1, 84<br>d. 1, 84
Teniposide<br>Cytarabina	100 mg/m²/i.v.<br>150 mg/m²/i.v.		d. 1–5 (28–32), (56–60)<br>d. 1–5 (28–32), (56–60)
**Maintenance Therapy**			
6-Mercaptopurine<br>Methotrexate	60 mg/m²/p.o.<br>20 mg/m²/p.o.	54–130	daily<br>once weekly

Abbreviations: [1] p.o., per os; [2] i.v., intravenously; [3] i.th., intrathecal; [4] d., day

Table S2. (**B**) GMALL 1/2004/T-LBL Total treatment duration in T-ALL was 2,5 years (with maintenance therapy), in T-LBL—52 months. After I consolidation (13 week), for patients who did not achieve CR/CRun., salvage therapy was applied (Nelarabine, FLAM-scheme), and allo-SCT in consolidation. In case of blood/bone marrow involvement, minimal residual disease (MRD) was monitored after induction I, induction II and consolidation. Patients with persistent MRD positivity (MRD > 10^{-4} after induction) and those with early and mature immunophenotype were transplanted at first complete remission. For T-ALL strategy based on monitoring MRD close to GMALL 07/2003. Central nervous system irradiation (24 Gy), after CR confirmation was recommended during second induction. Mediastinal irradiation (36 Gy) was not routinely recommended, it was considered only for patients with positive PET-CT after consolidation.

Drug	Dose	Week	Days of Administration
**Pre-Phase**			
Dexamethasone (Dx)<br>Cyclophosphamide<br>MTX/Dx	10 mg/m²/p.o.[1]<br>200 mg/m²/i.v.[2]<br>15/4 mg i.th.[3]	1	d.[4] 1–5<br>d. 3–5<br>d. 1
**Induction I**			
Dexamethasone<br>Vincristine<br>Daunorubicin<br><br>Peg-asparaginase	10 mg/m²/p.o.<br>2 mg/i.v./total<br>45mg/m² (30 mg > 55 ys[5])<br>1000 U/m²<br>(500 U > 55 ys)	2–4	d. 6–7, 13–16<br>d. 6, 13, 20<br>d. 6–7, 13–14<br><br>d. 20
**Induction II**			
Cyclophosphamide<br>Cytarabine<br>6-Mercaptopurine<br>MTX/Dx<br>Mediastinal irradiation (36 Gy) after phase II of induction, only for patients with PET-CT positive evaluation (individ-	1000 mg/m²/i.v.<br>75 mg/m²/i.v.<br>60 mg/m²/p.o.<br>15/4 mg i.th.	5–8	d. 26, 46<br>d. 28–31, 35–38, 42–45<br>d. 26–46<br>d. 28, 35, 42

ual decision) and prophylactic cranial irradiation (24 Gy) after achievement of CR			
	**Consolidation I**		
Dexamethasone	10 mg/m²/p.o.		d. 1–5
Vindesine	3 mg/m²/i.v. (max 5 mg)		d. 1
Methotrexate	1500 mg/m² i.v. 24 h (1000 mg/m² > 55 ys)	11–12	d. 1
Etoposide	250 mg/m²/i.v.		d. 4–5
Cytarabine	2000 mg/m² (every 12 h)		d. 5
MTX/Ara-C/Dx	15/40/4 mg i.th.		d. 12
	**Consolidation II**		
Methotrexate	1500 mg/m²/i.v. 24 h (1000 mg > 55 ys)	16–19	d. 1, 15
Peg-asparaginase	500 U/m²/ i.v.		d. 2, 16
6-Mercaptopurine	60 mg/m²/p.o.		d. 1–7, 15–21
	**Reinduction I**		
Prednisone	60 mg/m²/p.o.		d. 1–14
Vindesine	3 mg/m²/i.v. (max 5 mg)	24–26	d. 1,7
Adriamycine	50 mg/m²/i.v.		d. 1,7
MTX/Ara-C/Dx	15/40/4 mg i.th.		d. 1,15
	**Reinduction II**		
Cyclophosphamide	1000 mg/m²/i.v.		d. 15
Cytarabine	75 mg/m²/i.v.	24–26	d. 17–20, 24–27
6-Thioguanine	60 mg/m²/p.o.		d. 15–28
	**Consolidation III**		
Methotrexate	1500 mg/m²/i.v. 24 h (1000 mg > 55 ys)	30–33	d. 1, 15
Peg-asparginase	500 U/m²/i.v.		d. 2, 16
6-Mercaptopurine	60 mg/m²/p.o.		d. 1–7, 15–21
	**Consolidation IV**		
Teniposide	100 mg/m²/i.v.		d. 1–5
Cytarabine	150 mg/m²/i.v.	36–37	d. 1–5
MTX/Ara-C/Dx	15/40/4 mg i.th.		d. 1
	**Consolidation V**		
Cyclophosphamide	1000 mg/m²/i.v.		d. 1
Cytarabine	500 mg/m²/i.v. 24 h	41	d. 1
MTX/Ara-C/Dx	15/40/4 mg i.th.		d. 1
	**Consolidation VI**		
Methotrexate	1500 mg/m²/i.v. 24 h (1000 mg >55 ys)	46–48	d. 1, 15
Peg-asparginase	500 U/m²/i.v.		d. 2, 16
6-Mercaptopurine	60 mg/m²/p.o.		d. 1–7, 15–21
MTX/Ara-C/Dx	15/40/4 mg i.th.	52	d. 1
	**Maintenance Therapy**		
6-Mercaptopurine	60 mg/m²/p.o.	54–130	daily
Methotrexate	20 mg/m²/p.o.		once weekly

Abbreviations: [1] p.o.,per os; [2] i.v., intravenously; [3] i.th., intrathecal; [4] d., day; [5] ys, years old

Table S3. Baseline diagnostic characteristics of T-LBL patients (49 patients, including 2 with CD2 expression detected by immunohistochemistry).

	**Total** *n* = 49 (100%)
Gender, male	37 (75.5%)
Age, median (range)<br><35 year, *n* (%)	28 (17–56)<br>35 (71.4%)
mediastinal involvement<br>pleural effusion<br>pericardial effusion<br>abdominal/pelvis lymph nodes<br>nasopharynx<br>lung<br>kidneys<br>testis<br>breast<br>spinal canal infiltration	45 (91%)<br>23 (47%)<br>17 (34.7%)<br>8 (16.3%)<br>4 (8%)<br>1 (2%)<br>2 (4%)<br>1 (2%)<br>1 (2%)<br>1 (2%)
Bone marrow involvement<br>T-LBL (<25%)	 11 (22%)
CNS involvement	3 (6%)
Immunophenotype WHO 2008* [1]<br>Pro-T<br>Pre-T<br>Cortical/thymic<br>Mature/medullary<br>ETP**	9 (19%)<br>7 (15%)<br>28 (59.5%)<br>3 (6.5%)<br>9 (20%)
Median WBC (G/L)/(range)<br>HGB (g/dl)<br>PLT (G/L)	9.9 (2.5–21.2)<br>13.7 (9.1–17.7)<br>313 (151–788)
Treatment GMALL 05/93<br>GMALL 01/2004	20 (41%)<br>29 (59%)

Abbreviations: LBL, lymphoblastic lymphoma; CNS, central nervous system; ETP, early-T precursor; WBC, white blood count; HGB, hemoglobin; PLT, platelets; GMALL, German Multicenter Study Group for Adult ALL. * *n* = 47 patients, ** *n* = 45 patients.

Table S4. Clinical and immunophenotype characteristics, and outcome for T-LBL CD2(−) patients.

**No.**	**G/A**	**WHO2008/ WHO2017**	**CR**	**Clinical Outcomes**	**Allo-SCT**	**Status/Last FU (Years)**
1.	M/40	pro-T/ ETP	yes	R 15 mo after CR with massive BMinv.	no	DOD
2.	M/38	pro-T/ ETP	no	PD, mediastinal mass	no	DOD
3.	M/21	medullary/ non-ETP	yes	R 10 mo after CR with massive BMinv.	no	DOD
4.	M/38	medullary/ non-ETP	no	PD, mediastinal mass	no	DOD

5.	M/18	pro-T/ ETP	yes	R 11 ys after CR1 with BMinv., allo-SCT in CR2	yes CR2	ANED, CCR2 (5 ys)
6.	M/57	pro-T/ non-ETP	no	PD, CNSinv.	no	DOD
7.	M/30	pro-T/ ETP	yes	R 12 mo after CR with massive BMinv.	no	DOD
8.	F/30	pro-T/ ETP	yes	PD 2 mo after allo-SCT(CR1) with massive BMinv.	yes CR1	DOD
9.	M/39	pro-T/ non-ETP	no	PR, sepsis	no	TRM in PR
10.	M/29	pro-T/ ETP	yes	R 12 mo after CR, BMinv. with secondary AML	no	DOD
11.	M/19	cortical/ non-ETP	yes	R 18 mo after CR1 with soft tissue infiltration	yes CR2	ANED, CCR2 (11 ys)
12.	M/23	pro-T/ non-ETP	yes	CR, allo-SCT in CR1	yes CR1	ANED, CCR1 (3 ys)
13.	M/24	cortical/non-non-ETP	yes	R 2 mo after CR, CNSinv with PMRinv in FCM	Yes CR2	ANED CCR2 (3 ys)

Abbreviations: No., case number; G/A, gender/age (y), M-male, F-female; R, relapse; CR, complete remission; CCR, continuous complete remission; PR, partial remission; PRpd, Partial Remission followed by progressive disease; PD, progressive disease; BMinv, bone marrow involvement; CNSinv, central nervous system involvement; TBI, total body irradiation; SCT, stem cell transplantation; * autologous bone marrow transplantation; mo, months; ys, years; FU, follow up years after the end of treatment; ANED Alive, no evidence of disease; DOD, Died of Disease progression; TRM, Treatment Related Mortality; FCM, flow cytometry.

Table S5. Immunophenotype details (WHO 2008 [1] T-ALL/LBL subtypes).

**Subtype (WHO 2008)**	**Total** *n* **= 71 (100%)**	**T-ALL** *n* **= 26 (37%)**	**T-LBL** *n* **= 45 (63%)**	***p***
Early pro-T	15 (21%)	6 (23%)	9 (20%)	0.76
Early pre-T	13 (18%)	6 (23%)	7 (16%)	0.43
Cortical/thymic	38 (54%)	12 (46%)	26 (58%)	0.344
Medullary/mature	5 (7%)	2 (8%)	3 (7%)	0.871
ETP (WHO 2017)	13 (18%)	4 (15%)	9 (20%)	0.598

Abbreviations: ALL-acute lymphoblastic leukemia; LBL-lymphoblastic lymphoma, WHO-World Health Organization, ETP-early T-cell precursor.

Reference:

1. Borowitz, M.J.; Chan, J.K.C. T lymphoblastic leukaemia/lymphoma. In *WHO Classification of Tumours of Haematopoietic and Lymphoid Tissues*, 4th ed.; Swerdlow, S.H., Campo, E., Harris, N.L., Eds.; IARC: Lyon, France, 2008; pp. 176–178
